# Dual-tasking while using two languages: Examining the cognitive resource demands of cued and voluntary language production in bilinguals

**DOI:** 10.1177/17470218231173638

**Published:** 2023-05-18

**Authors:** Angela de Bruin, Ronan McGarrigle

**Affiliations:** 1 Department of Psychology, University of York, York, UK; 2 Department of Psychology, University of Bradford, Bradford, UK

**Keywords:** Bilingualism, language switching, dual-tasking

## Abstract

The way bilinguals switch languages can differ depending on the context. In cued dual-language environments, bilinguals select a language in response to environmental cues (e.g., a monolingual conversation partner). In voluntary dual-language environments, bilinguals communicating with people who speak the same languages can use their languages more freely. The control demands of these types of language-production contexts, and the costs of language switches, have been argued to differ (Adaptive Control Hypothesis). Here, we used a dual-task paradigm to examine how cued and voluntary bilingual production differ in cognitive resources used. Forty Mandarin-English bilinguals completed two language-switching paradigms as the primary task; one in response to cues and one while using two languages freely. At the same time, they also had to respond to the pitch of tones (secondary task). Response times (RTs) on the secondary task, as well as naming times on the primary task, were shorter under the voluntary- than cued-naming condition. Task workload ratings were also higher under the cued- than voluntary-naming condition. This suggests more attentional resources are needed in a cued-naming context to monitor cues and select languages accordingly. However, the costs associated with switching from one language to the other were similar in both voluntary- and cued-naming contexts. Thus, while cued-naming might be more effortful overall, cued and voluntary *switching* recruited similar levels of cognitive resources.

## Introduction

At least half of the world’s population can hold a conversation in more than one language (Grosjean, 2010). For these bilingual speakers, language production includes not just selecting which *words* to use but also which *language* to produce them in. In addition, bilinguals can switch languages, although the frequency and type of switching depend on the type of language environment they operate in (Green & Abutalebi, 2013). Much previous research has focussed on language contexts that dictate which language has to be used and when bilinguals need to switch. For example, a Mandarin-English bilingual will need to use English when communicating with an English monolingual speaker. In this case, there is a specific “cue” (i.e., the presence of an English speaker) that indicates which language has to be used and, in some cases, when a language switch is needed (e.g., when going from a Mandarin phone call to talking with this English speaker). This type of language switching has been studied frequently in so-called cued language-switching paradigms, in which bilinguals typically name objects depicted in pictures in the language represented by a visual cue (e.g., a country flag). However, this is not the only type of language switching bilinguals can engage in. When surrounded by other bilinguals who speak the same languages, a bilingual might be able to use both languages more freely and switch for other reasons, like when a word simply comes to mind faster in another language. Research has started to examine the cognitive mechanisms behind this type of language switching and potential differences between “cued” and “voluntary” switching. One of the main open questions concerns the amount of cognitive effort or attentional resources that underlie these two types of language production. For example, does using two languages freely and switching between them recruit fewer attentional resources than switching in response to cues? In this study, we addressed these questions through a dual-tasking paradigm in which participants responded to high- or low-pitched tones while naming pictures in their language of choice or in response to cues.

### Cued language production

Language switching during production has predominantly been studied by asking participants to name digits or pictures in response to cues. For example, Mandarin-English bilinguals might be presented with a country flag instructing them to name pictures in Mandarin or English. Within a dual-language context, both languages need to be used interchangeably (i.e., some pictures are presented with a Mandarin cue and some with an English cue). Some trials will require a switch between languages (“switch” trials), while other trials allow for the same language to be used as in the previous trial (“non-switch” trials). Comparing response times (RTs) typically shows that bilinguals need more time to name pictures in switch than non-switch trials (“switching cost,” e.g., Costa & Santesteban, 2004; Meuter & Allport, 1999). These switching costs are argued to reflect a more reactive type of language control that might combine both language activation as well as inhibition processes. When switching languages, bilinguals need to activate the new target language (e.g., Mandarin). However, they also need to make sure they do not accidentally use the other, non-target language that they used in the previous trial (e.g., English). Bilinguals might apply inhibition over the current non-target language to achieve this. In addition, part of the switching costs might reflect the need to overcome previously applied inhibition (Green, 1998). Switching from English to Mandarin might not just involve applying inhibition over English but also lifting Mandarin inhibition applied in the previous trial (i.e., when naming in English). Some explanations of language-switching costs focus more on activation than inhibition, with switching costs argued to stem from over-activation of a target language, which can slow down responses on the next trial when a switch is needed (e.g., Philipp et al., 2007). However, both activation- and inhibition-based accounts argue that cued language switching requires additional effort, as compared to non-switches, in the form of reactive language control.

In addition to more reactive control processes, cued language use also requires proactive control. Comparisons between cued dual-language switching contexts and single-language contexts (in which bilinguals name all pictures in the same language) typically show slower responses in the dual- than the single-language context. This is known as a “mixing cost” (e.g., Christoffels et al., 2007). Crucially, this difference is assessed by comparing single-language trials to the *non-switch* subset of dual-language trials only, thus ruling out the influence of language switch trials. Rather, this mixing cost is taken to reflect the more sustained control needed when using two languages in response to cues. In this case, bilinguals constantly need to monitor cues in their surroundings to determine which language to use. Goal maintenance is needed to actively keep languages and rules in mind, to make sure the appropriate language is selected at the right moment (i.e., in response to the cue), and to monitor conflict between the two languages. To achieve this, bilinguals might proactively balance the relative activation of each language to facilitate the flexible use of two languages, either by increasing activation of the less proficient language or by inhibiting the more proficient language (e.g., Christoffels et al., 2007).

### Proactive and reactive control demands across interactional contexts

Proactive and reactive control demands might, however, depend on the type of language-switching environment. The Adaptive Control Hypothesis (Green & Abutalebi, 2013) distinguishes between language switching in more controlled dual-language contexts and switching in free code-switching environments. Dual-language switching environments are most similar to the cued switching tasks described above. For example, a Mandarin-English bilingual working in the United Kingdom might use both languages at work but English with English monolingual colleagues and Mandarin with Mandarin-speaking colleagues. In this context, language switching occurs but only when indicated by cues in the context. For example, this bilingual might switch from Mandarin to English when an English-speaking colleague enters the room. As described above, this context might require reactive control processes in response to the actual stimulus (e.g., task engagement, task disengagement, and reactive inhibition; Green & Abutalebi, 2013; see also Braver, 2012). In addition, bilinguals might use a series of control mechanisms not just reactively but also proactively to prepare in advance for the task of using two languages and to manage competition and interference (e.g., Declerck, 2020). These proactive control mechanisms can include sustained goal maintenance, conflict monitoring, managing global interference between languages, and detecting cues to select the corresponding target language (e.g., Green & Abutalebi, 2013).

However, other language contexts might allow for more free switching that occurs within sentences too. For example, when the same Mandarin-English bilingual is speaking with friends who all speak both languages, both languages can be used interchangeably without the restrictive factor of interlocutors only understanding one of the languages. In these free-switching environments, bilinguals might switch between languages for a range of reasons. One of the key reasons might be a lexical access-driven language choice, such that bilinguals use a word in a certain language because it is retrieved faster in that language than in the other languages (e.g., de Bruin et al., 2018; Sarkis & Montag, 2021). Related to this, bilinguals might also use words in a certain language because they prefer a certain language for a given topic or concept (e.g., de Bruin & Martin, 2022) or simply because no translation equivalent exists in the other language.

According to the Adaptive Control Hypothesis (Green & Abutalebi, 2013), dual- and free-switching environments differ in terms of both proactive and reactive control. In a free-switching environment, there might be less need for proactive control mechanisms to maintain information related to cues and language goals in mind, when both languages are understood by all speakers. Furthermore, less reactive control might be needed if language switching is driven by opportunistic use, with bilinguals choosing and switching languages because lexical access is faster in another language. However, while competitive control might not be needed, control might not be suspended entirely, especially when switches are alternations between languages or insertions of one word. When referring to a specific object, unimodal bilinguals using two spoken languages can still only use one word at a time. A more cooperative form of control might still be used to ensure the two languages can be used within the same context. In this case, local control over language production could flexibly shift between the two languages depending on the words that can be retrieved faster or are preferred (Green & Wei, 2014).

Furthermore, language choice and switching are not driven entirely by variables related to lexical access but may also be influenced by strategic, emotional, or ideological choices (Ritchie & Bhatia, 2012). Language choice can also be influenced by primes in the context that are more aligned with one language, including visual information such as flags or lanyards (e.g., de Bruin & Martin, 2022; Vaughan-Evans, 2023) as well as the language behaviour of the person they are interacting with (e.g., Fricke & Kootstra, 2016; Kootstra et al., 2020). This suggests that even during free-switching environments, language control might be less needed but not entirely suspended.

### Voluntary language production

To assess language control in contexts more similar to free-switching contexts, some studies have compared cued to voluntary-naming tasks. While cued-naming tasks use cues to instruct bilinguals which language to use for each picture, voluntary-naming tasks typically just instruct bilinguals to name each picture in their language of choice. Bilinguals are usually instructed that they can use both languages and switch freely when they want. Similar to cued tasks, these free contexts can also be compared to single-language contexts.

#### Proactive control

In terms of proactive control that is applied across switch and non-switch trials, two main comparisons between conditions have been used to assess the underlying mechanisms during voluntary naming. The first concerns a comparison in overall reaction times with cued picture-naming tasks, which typically reveals faster responses in voluntary- than cued-naming tasks (e.g., de Bruin et al., 2018, 2020; Gollan et al., 2014; Jevtović et al., 2020). These faster voluntary- than cued-naming responses do not just reflect a difference in overall task demands. Jevtović et al. (2020) compared mandatory and voluntary naming within the same context. On each trial, Spanish-Basque bilinguals saw a picture with either a Spanish or Basque flag (mandatory language choice) or a cue combining both flags (voluntary language choice). Even within this context, which demanded cue detection and overall goal maintenance for both mandatory and voluntary naming, RTs were shorter on voluntary trials. The second measure used to capture proactive control differences between cued and voluntary switching is a comparison of mixing effects. While cued naming studies consistently reveal mixing costs (e.g., Christoffels et al., 2007; de Bruin et al., 2018), voluntary tasks often show mixing benefits in either both languages (e.g., de Bruin et al., 2018, 2020; Grunden et al., 2020) or in one of the languages (e.g., de Bruin & Xu, 2023; Gollan & Ferreira, 2009). These voluntary benefits are at least partly driven by speed of lexical retrieval during free naming (de Bruin et al., 2018). Production in this context might be faster as words that are particularly slow to be retrieved in one language might be avoided and named in the other language instead, which cannot be done in cued language or single-language contexts. These findings also suggest that voluntary naming might require less proactive control than cued naming or even single-language contexts. When both languages are possible targets, faster naming might be possible due to lower proactive control demands in terms of goal monitoring and proactively controlling interference from a “non-target” language.

#### Reactive control

With respect to trial-by-trial switching costs, and the reactive control associated with these, findings are more mixed. Many studies have found that switching voluntarily still comes with a switching cost, with bilinguals taking more time when switching languages than when continuing in the same language. This cost has been observed in several voluntary picture-naming studies (e.g., de Bruin et al., 2018, 2020; de Bruin & Xu, 2023; Gollan et al., 2014; Gollan & Ferreira, 2009; Gross & Kaushanskaya, 2015) as well as in corpus studies capturing natural language use (e.g., Fricke et al., 2016). Some find these switching costs to be of the same size as cued costs (e.g., de Bruin et al., 2018; Experiment 1 in Gollan et al., 2014), while others find the cost to be smaller in voluntary than in cued switching (e.g., de Bruin & Xu, 2023; Experiment 2 in Gollan et al., 2014; Jevtović et al., 2020; and see Blanco-Elorrieta, & Pylkkänen, 2017, for an example of no voluntary switching costs).

While, as discussed above, lexical access is one of the main mechanisms in voluntary language use, the presence of switching costs suggests that there might still be some degree of reactive control. Even when switching voluntarily, and potentially similar to cued switching, bilinguals might recruit some reactive control to make sure a target word is produced in *one* language only (as it is impossible to use both languages at once). This could take place in different forms. Switching costs might reflect bilinguals needing to apply some reactive inhibition over the new non-target language to facilitate a switch. Even when switching for purely lexical-access reasons (e.g., because “horse” is produced faster in Mandarin than in English), just having used the other language might lead to some ongoing competition from the previous trial that needs to be resolved (potentially through inhibition). Similar to cued costs, voluntary switching costs might also reflect time needed to overcome previously applied reactive inhibition. For example, a bilingual might be faster to name “horse” in Mandarin than in English and might therefore switch languages, but if there was some reactive inhibition of Mandarin in the previous trial, additional time might be needed to overcome this. Other explanations do not necessarily involve inhibition or reactive control. Baseline activation levels might differ on each trial depending on which language was just used. When using English, the activation level of words in this language might (temporarily) increase. When switching to Mandarin, even if the Mandarin word is accessed faster than the English word or preferred, the relative difference in activation might be smaller during a language switch than during a non-switch, creating more overall competition and potentially slowing down access to the Mandarin word. Thus, language competition and coordination might continue to play a role in voluntary language switching rather than being suspended entirely (cf. Green & Wei, 2014), although it is unclear whether this is in the form of voluntary switching recruiting reactive (potentially inhibitory) control, similar to cued switching (e.g., Goldrick & Gollan, 2023).

Furthermore, bilinguals can differ in how strongly language choice is driven by lexical access. When participants are encouraged to use a purely bottom-up, lexically driven approach to language choice, switching costs are not always found. Kleinman and Gollan (2016) enforced a lexically driven approach to language choice by instructing bilinguals to always name a given picture in the same language. Upon seeing a picture for the first time, bilinguals had to decide which language they wanted to use for that item and then had to consistently name that picture in that language throughout the task. Switching costs were absent in this type of switching while they were present in voluntary-switching tasks without further instructions and in cued-switching tasks (Kleinman & Gollan, 2016). Absent switching costs have also been found when items are used that are more closely related to one language/culture than the other. For example, in a study with Australian English-Mandarin bilinguals, Zhu et al. (2022) found no switching costs when using items such as “wombat” that are more routinely used in the Australian English language than in Mandarin. In this instance too, language choice is pushed to be consistent for each item and to be lexically driven as the picture name is slow to retrieve or is not often used at all in the other language. Finally, some studies (e.g., Gollan et al., 2014) have suggested switching costs might be related to individual differences *between* bilinguals in terms of their language choice. Bilinguals who used the same language more consistently for a given item were found to have smaller switching costs. This is in line with the approach described above in Kleinman and Gollan (2016), although now observed in terms of individual differences that occur naturally. Bilinguals that use more bottom-up, lexical-access-driven naming approaches might not show switching costs while bilinguals that are more susceptible to other influences (e.g., the word they used on the previous trial or a general preference for one of the languages) may need to apply more control and therefore show costs. However, these patterns are not always observed and some studies show switching costs even in bilinguals who use a lexical-access-driven naming approach (e.g., de Bruin et al., 2018). Results with respect to switching, and associated reactive control mechanisms, thus remain very mixed.

### Dual-tasking

To better understand the attention demands of cued and voluntary naming, as well as how they may or may not differ in terms of reactive and proactive control, we used a “dual-task” paradigm. Dual-task paradigms have long been used in experimental psychology to assess attentional resource demands of a variety of cognitive processes (Kahneman, 1973; Pashler & Johnston, 1998). In this type of paradigm, participants are asked to complete two tasks at the same time. In the domain of language production, participants have, for example, been asked to name pictures (primary task) while also responding to tones (secondary task; e.g., Ferreira & Pashler, 2002; Piai & Roelofs, 2013). The dual-task paradigm relies upon the assumption that there is a finite pool of attentional resources that can be used when performing multiple tasks simultaneously. As a result, if one task (i.e., the primary naming task) requires an increase in resource allocation, this will become evident as an RT decrement in the secondary (i.e., tone discrimination) task. In other words, when a primary task requires more effort, fewer resources are available for the secondary task and performance should decrease (reflected in longer RTs). RTs on the secondary task can then be compared across different primary task (e.g., cued versus voluntary naming) conditions. Several studies have suggested that secondary task performance can indeed be influenced by the cognitive demands of the primary speech production task (e.g., Fournet et al., 2021; Piai & Roelofs, 2013).

Piai and Roelofs (2013) asked participants to name pictures while seeing words that were either the same word as the picture name (e.g., picture of a dog and word “dog”), unrelated words (e.g., “table”), or semantic distractors (e.g., “cat”). While doing this, participants were presented with tones and asked to indicate with a button press whether they heard a high- or low-pitched tone. Responses on the secondary tone-discrimination task were sensitive to changes in the difficulty level of the primary naming task, with slower tone-discrimination responses shown during semantic distractor trials. In Fournet et al. (2021; Experiment 1), participants were asked to perform a verbal semantic fluency (primary) task while executing a “go/no-go” (secondary) task. Primary task difficulty was modulated by using relatively “easy” naming categories (e.g., animals and clothes) and relatively “hard” naming categories (e.g., sports and jobs) based on pilot testing. Secondary task performance decrements were shown for the hard compared to the easy category condition in terms of both poorer performance accuracy and longer RTs. Results from both studies suggest that the dual-task paradigm can be used to assess the cognitive resource demands involved in language production.

### Current study

In the current study, we created a dual-task paradigm in which participants had to press buttons to discriminate between tones (high/low pitch) while also naming pictures in either a cued or voluntary naming task. In the cued task, participants named pictures in the language corresponding to the country cue while the voluntary task asked them to name the pictures freely in Mandarin and English. We focussed on RTs in the secondary tone-discrimination task as the primary outcome measure. Secondary tasks during the dual-task paradigm can take a variety of forms that impose relatively complex demands (e.g., go-no go, Fournet et al., 2021) versus more basic perceptual requirements (e.g., tone discrimination, Ferreira & Pashler, 2002). We opted to use a tone-discrimination task as this type of task has been used in previous dual-task paradigms with language production (e.g., Ferreira & Pashler, 2002). Furthermore, tone discrimination does not require language or high-level control. This allowed us to study the influence of the primary task demands on the secondary task while minimising the influence of the secondary task on the primary production task.

Using a dual-task paradigm offered two key benefits over purely assessing language production in a single-task paradigm. First, dual-tasking taxes the cognitive system more strongly than completing a single task (e.g., just language production; Ferreira & Pashler, 2002). More subtle differences between cued and voluntary naming might be more likely to emerge under these more taxing conditions. Second, it allowed us to better understand *why* cued and voluntary tasks both show switching costs. It remains largely unclear if both cued and voluntary switching costs reflect similar language control mechanisms used by bilinguals to manage competition between languages.

If cued and voluntary switching are equally effortful and recruit the same amount of control resources, we would expect tone-discrimination switching costs (longer tone-discrimination RTs during language switch than non-switch trials) to be comparable during cued and voluntary switching. However, if cued and voluntary switching differ in reactive control, we expect a larger tone-discrimination switching cost during cued than voluntary naming. This might occur even if the actual naming costs are similar for the cued and voluntary task. Voluntary *naming* switching costs might reflect processes that take additional time but that do not necessarily require (the same amount of) cognitive control resources. For instance, persisting activation of language A on trial *n* − 1 could increase the time needed to use and switch to language B on trial *n*, even if no additional control is used or required. Thus, if voluntary switching does not use (the same amount of) reactive control in the same way as cued switching, differences might emerge in the secondary task, even if they do not emerge in the naming data.

In terms of proactive control, if cued and voluntary naming differ, we would expect the cued task to leave fewer attentional resources for the secondary task. In this case, tone-discrimination RTs should be longer (poorer performance) in the cued than the voluntary naming task. These proactive control differences should affect overall RTs, including non-switch trials. Similar tone-discrimination RTs (i.e., no main effect of task) would suggest similar resource demands for both cued and voluntary naming. Finally, cognitive effort can be measured in a number of different ways, including subjective, behavioural (e.g., dual-task paradigm), and physiological indices, with each measure thought to tap related, but disparate, processes (Alhanbali et al., 2019; Pichora-Fuller et al., 2016). To examine whether participants experienced changes in perceived effort as a function of naming condition (cued versus voluntary), we also administered the NASA task load index (Hart & Staveland, 1988) after each condition. Consistent with the dual-task paradigm, we predicted that participants would report greater perceived workload in the cued compared to the voluntary-naming condition.

## Methods

The study’s pre-registration and data and analysis scripts can be found at https://osf.io/2kftd/.

### Participants

The study received ethical approval from the Ethics Committee in the Department of Psychology at the University of York and all participants provided written informed consent. The study was completed by 40 Mandarin-English bilinguals. All participants were invited using Prolific (Prolific.co). A short pre-screening was completed by 137 participants. This pre-screening was used to select participants who met our recruitment criteria of being a native speaker of Mandarin living in the United Kingdom or United States, without hearing or vision problems (including colour blindness). We also used the pre-screening to ensure participants could record their audio responses well. Seventy-three participants met the criteria and were invited to take part in the study. Of the 66 participants who started the study, 11 participants could not be included due to issues with the recordings (e.g., no, empty, or very noisy recordings); nine participants were excluded as they were not able to play the tones; four participants did not respond to the invitation for the second session; and two participants named each picture in both languages in the voluntary-naming task. These participants were excluded, leading to a final sample size of 40 participants. This sample size was determined in two ways. First, as a rule-of-thumb, a minimum of 1,600 observations per condition has been suggested to reach adequate power in mixed-effects analyses (Brysbaert & Stevens, 2018). The cued task includes 60 trials per condition (i.e., per combination of task, language, and trial type), yielding 2,400 observations per condition with 40 participants. In the voluntary task, the number of trials per condition differs (depending on the language used on each trial), but 240 trials should yield a good number of trials per condition for all participants (and indeed, as a mean across participants, each condition included at least 37 trials in the RT analysis after removal of incorrect responses and RT outliers). Second, we ran a pilot study with five participants to determine the impact of cued language switching on the secondary task RTs in the dual-tasking paradigm. Based on the switching-cost effect size observed (*d* = 1.2), 40 participants provided sufficient power to detect an effect of language switching in the tone-discrimination task.

All participants (*M age* = 26.3, *SD* = 5.3; 29 female) were native speakers of Mandarin. They completed a language background questionnaire and a short typed picture-naming task (based on de Bruin et al., 2017) to assess English vocabulary (see Table 1). Most participants acquired English during childhood (*M* start age of English acquisition = 7.2, *SD* = 3.5, range = 0–13 years old). All participants had been living in the United Kingdom or United States for at least a year prior to testing (seven participants were born there). They reported normal or corrected-to-normal vision and hearing and no known neurological or reading difficulties. All apart from three were right-handed. Eleven participants reported (some) knowledge of another language in addition to Mandarin and English, in most cases Cantonese. While participants were native speakers of Mandarin, they had high proficiency in English too and, on average, were using English more in their daily lives than Mandarin (see Table 1). All participants reported switching languages on a daily basis (*M* = 2.6, *SD* = 1.1, on a scale from 1 = *switching all the time* to 5 = *not switching at all*), and occasionally switching within a conversation (*M* = 3.2, *SD* = 0.9) and within a sentence (*M* = 3.3, *SD* = 0.9).

**Table 1.** Summary of the participants’ language background.

	Mandarin—*M* (*SD*)	English—*M* (*SD*)
Picture-naming vocabulary (0–65)	Not assessed	62.0 (4.3)^a^
Self-rated proficiency (0–10)		
Speaking	9.0 (1.3)	8.5 (1.6)
Understanding	9.3 (1.0)	8.9 (1.0)
Writing	7.2 (3.1)	8.2 (1.5)
Reading	8.1 (2.8)	8.9 (1.1)
Daily-life use (0%–100%)	37.3 (27.6)	65.7 (27.5)

Proficiency was assessed through self-ratings in both languages and through a picture-naming vocabulary task in English. Language use was assessed through a self-rated scale asking participants to indicate with a percentage from 0 (*never*) to 100 (*always*) how often they used Mandarin and English on a daily basis.

a All participants scored above 40 points, the cutoff point for inclusion in our pre-registration.

### Tasks and design

In two separate sessions, to avoid the influence of one naming task on the other, participants named pictures in either a cued or a voluntary naming task (primary task) while also responding to the pitch of a tone (secondary task). They were instructed to indicate the pitch of the tone with a button press. Our main dependent variable was the tone-discrimination RT. We also recorded and analysed naming times (i.e., the onset of language production in response to each picture).

Independent variables (all within-subject) included Task (cued or voluntary naming), Trial type (language switch or non-switch), and Language (Mandarin or English). In the cued task, participants were instructed to name the picture in the language corresponding to the cue. In the voluntary task, participants were free to choose the language for each picture. Trial type was defined as a switch when the language used to name the current picture differed from the previous picture and as a non-switch if the same language was used as in the previous picture. Language and trial type were determined by the language cue in the cued task and by the participants’ free naming language in the voluntary task.

### Materials

Twenty pictures were selected from the MultiPic database (Duñabeitia et al., 2018) that participants named in both the cued and voluntary task (see the online Supplementary Material). Pictures represented easy-to-name objects (e.g., animals or food) corresponding to high-frequency words in both languages. English words were between one and five phonemes and one or two syllables long. Mandarin words consisted of one or two characters. ZIPF frequency (SUBTLEX-CH for Mandarin, Cai & Brysbaert, 2010; SUBTLEX-UK for English, Van Heuven et al., 2014) did not differ significantly between English (*M* = 4.8, *SD* = 0.4) and Mandarin (*M* = 4.6, *SD* = 0.6; *t*(19) = −1.467, *p* = .159), translation equivalents.

For the cued task, two versions of each country flag were used to indicate which language had to be used to name the picture. Two cue versions alternated to avoid a confound between cue and language switching influencing switch costs (e.g., Heikoop et al., 2016). This way, both language switch and non-switch trials were always a cue switch in the cued and voluntary task, thus minimising differences between the two tasks. Participants living in the United Kingdom saw the British and Chinese flag (one version of each language cue was in the form of a flag and the other one used the colours of the flag presented in the shape of the country). Participants living in the United States saw the American and Chinese flags. In the voluntary task, we created cues that were a combination of both flags/countries, to indicate that participants could name the picture in their language of choice. This ensured that participants received the same type of cues and visual input in both the voluntary and cued task. Furthermore, we chose cues naturally associated with each language to avoid participants having to memorise cue-language pairings, which could have introduced differences between the cued and voluntary tasks.

The NASA task load index (Hart & Staveland, 1988) was used as a measure of self-reported mental workload. The NASA task load index is a widely used subjective measure of the cognitive demands of language processing (McGarrigle et al., 2021; Pichora-Fuller et al., 2016; Strand et al., 2018). In this questionnaire, participants were first asked to indicate how they experienced the task in terms of mental demand (how mentally demanding was the task?), physical demand (how physically demanding was the task?), temporal demand (how hurried or rushed was the task?), performance (how successful were they in accomplishing the task?), effort (how hard did they have to work to achieve that performance?), and frustration level (how insecure, discouraged, irritated, stressed, and annoyed were they)? They were asked to think about the dual-tasking part when providing these ratings on a slider from 0 (*very low*) to 100 (*very high*). Next, they were asked to indicate which of the above six experiences they found more important when describing the experienced workload. They were given every possible combination (e.g., performance and physical demand) and were asked to select the most important one for each comparison.

### Procedure

Participants first completed a pre-screening to make sure they met our language background criteria (see section “Participants”). They also named a few pictures to make sure their naming responses could be recorded. Participants who were invited to take part in the study completed two sessions (*M* interval in days = 8.4, *SD* = 7.2, range = 3–35 days), in which they either completed the cued or voluntary naming task, with the order counterbalanced across participants. The study was run online on Gorilla.sc (Anwyl-Irvine et al., 2020).

At the start of each session, participants completed a headphone check. They were asked to wear headphones with a microphone and to complete the study in a quiet environment. In the headphone check, participants heard three sounds and had to indicate with a button press which sound was the quietest. This task was set up so that it could only be completed correctly with the use of stereo headphones (see Woods et al., 2017, for details). Participants completed six trials and had to get at least five correct to continue with the study. They were given two attempts to achieve this. Next, participants completed a sound check to make sure the tones could be presented and to allow them to adjust their volume where necessary. Participants had to indicate whether the tone was “high” or “low” and could only continue if they gave the correct answer. Finally, participants completed a microphone check in which they were asked to record a word and listen back to their own recording. They were asked to continue only if they could hear their recording clearly.

Next, the dual-task started. First, participants completed the tone-discrimination task only (single-task) in which they were asked to indicate whether a tone had a high or low pitch. This started with four practice trials, followed by 40 experimental trials. Participants pressed “1” in response to a *low tone* and “2” in response to a *high tone*, with these instructions remaining on the screen while participants were responding to the tones. High-pitch tones were 1,000 Hz and low-pitch tones 400 Hz. There was no time limit per trial and the next trial started as soon as a response was given. Trials were separated by a 500-ms interval presenting a fixation cross.

After the tone-discrimination-only task, participants were familiarised with the pictures and corresponding words in both languages. They saw one picture on the screen at a time, with the Mandarin and English words, and were asked to just look at the picture and read the words in silence before pressing space to see the next picture and words. They then completed a single-language block in each language in which they named all pictures once in English and once in Mandarin, and the order of language blocks was counterbalanced across participants. Each single-language block was preceded by four practice trials. Prior to seeing the picture, participants saw a fixation cross for 500 ms followed by the language cue for 300 ms. Next, the picture was presented for 2,500 ms with the cue. The picture stayed on the screen for a fixed duration regardless of when a response was given. Participants next completed a short language-switching practice, in which they saw 12 trials that required them to either name the picture in response to the cue (cued) or in their language of choice (voluntary). Pictures used during the practice phases differed from those used in the experimental parts.

After practicing the primary and secondary tasks separately, participants then practised the two tasks together (eight trials), followed by the 240 experimental dual-task trials. In the cued task, participants were instructed to name the picture in the language matching the country cue. In the voluntary task, participants received the following instructions: “In the next task, you are free to name each picture in your language of choice (Mandarin or English). Before each picture, you will see a combined [British/American]/Chinese flag. This flag tells you that you can freely choose your naming language. For each picture, use the word that comes to mind fastest regardless of the language. Just name the picture in English OR in Mandarin. You can switch languages when you want. Name the picture as fast as you can without making mistakes. Make sure to sometimes use Mandarin and sometimes English in this task.” For simplicity (to reduce the amount of text on the screen), instructions were given in English only. In both task instructions, the naming instructions were followed by instructions about the tone-discrimination task. Participants were asked to complete both tasks accurately and quickly but to prioritise the naming (primary) task. The temporal overlap in this task between planning of word production and auditory discrimination draws upon working-memory resources, needed to ensure successful completion of both tasks in a relatively short time window. The task was set up this way to increase sensitivity to potential differences in the secondary task related to the cognitive effort needed in the primary task.

**Figure 1.**
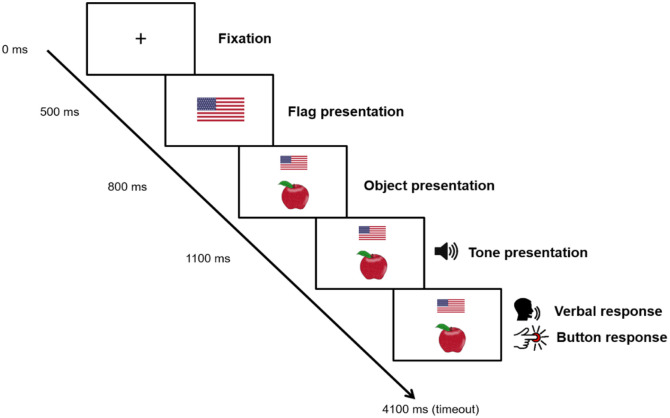
Schematic overview of a trial. In this example, we show a cued trial in which a picture of an apple had to be named in English by participants living in the United States.

Each dual-task trial started with a fixation cross for 500 ms, followed by the presentation of the language cue for 300 ms in the middle of the screen (see Figure 1 for an overview of a trial). Then, the picture and cue were presented together (with the cue above the picture) for 3,300 ms. The tone was presented 300 ms after the onset of picture presentation. The next trial always started 3,300 ms after picture presentation, regardless of when a response was given. Breaks were given after every 80 trials. Within the cued task, half of the trials were language switches (i.e., different languages than in the previous trial) and half were non-switches (i.e., the same language as on the previous trial). Half of the trials of each type were presented in Mandarin and the other half in English. There were no more than four trials of the same type in a row. Each picture occurred an equal number of times in each combination of trial type and language and lists were pseudo-randomised so that the same picture did not appear twice in a row. In the voluntary task, trial type and language were dependent on the participants’ responses and coded afterwards. Each task included 240 trials, with each picture being repeated 12 times in each task.

After the dual task in each session, participants completed the NASA-TLX (Hart & Staveland, 1988). At the very end of the second session, participants also completed the language background questionnaire and the short picture-naming vocabulary task (see section “Participants”). We also asked them after each session if they experienced any issues; a few participants indicated problems playing the tones correctly and were removed (see section “Participants”). Each session took approximately 30 to 45 min to complete.

### Data analysis

The primary outcome variable, as pre-registered, was RTs during the tone-discrimination task (secondary task). We used linear mixed-effect models using lme4 package (version 1.1-30) and lmerTest (version 3.1.-3) in R 4.2.1 to analyse these data. Accuracy was close to the ceiling (see section “Results”) and not analysed further. Prior to the RT analysis, we removed trials with an incorrect tone response or a picture-naming error. These removals included: no response at all, a response in the wrong language (cued task only), a wrong name for the picture (e.g., “horse” instead of “cat”), or a response that combined both languages. We also removed trials preceded by a trial with a picture-naming error as these could not be classified as either switch or non-switch trials (with the exception of trials that were named in the correct language but used the wrong word). For the same reason, trials preceded by a break were not included in the analysis. RT outliers (3 *SD* above/below mean per participant and language/trial type/task) were removed using *trimr* (Grange, 2015). This resulted in the removal of 0.7% of included trials after accuracy removal. Visual inspection of histograms and q–q plots showed tone RTs were normally distributed and they were therefore analysed without log transformation.

Analyses started with a maximal structure including participants’ and items’ intercepts and slopes. When a model did not converge, we first removed correlations between intercepts and slopes and then by-item slopes that explained the lowest amount of variance. The model included the fixed effects of Language (Mandarin coded as −.5 and English as .5); Trial type (non-switch = −.5; switch = .5); and Task (voluntary = −.5; cued = .5); and their interactions. Our pre-registration included mean RT on the single-task tone discrimination as a potential predictor. We indeed included this predictor to account for the individual differences observed in terms of tone-discrimination responses. Analyses not including this predictor, however, showed the same effects. Given that each session started with its own single-task tone discrimination block of trials, we included one single-task tone-discrimination mean for the voluntary session and one mean for the cued session per participant. It was scaled and centred and included as a fixed main effect only (i.e., not interacting with other variables). The model converged after removal of the item slopes for trial type, task, and task × trial type. Before data collection, we were unsure whether the participants’ recordings would be of sufficient quality to analyse the actual picture-naming data. We therefore focused on secondary-task analyses in the pre-registration. However, recordings were of good quality and we therefore also analysed their naming data (primary task RTs). Naming RTs were determined from the recordings using CheckFile in Checkvocal (Protopapas, 2007), which provides an automatic indication of naming onset that was checked manually and adjusted where necessary. The analysis followed the same process as described above for the tone discrimination data. Visual inspection of histograms and q–q plots showed naming data were not normally distributed and they were therefore log-transformed. The (log) RT outlier process removed 0.6% of trials.

We also assessed the NASA task load index data reflecting the overall experience of workload for the cued and the voluntary tasks. As recommended in the scoring procedure (Hart & Staveland, 1988), these scores were weighted by considering which experiences participants valued most. Each experience received a weight of 0 to 5, depending on how often a participant selected the experience in the value comparisons. The participant rating for each of the six experiences was then multiplied by the corresponding weight. These ratings were summed and then divided by 15 (the sum of the weights). A paired t-test was conducted to compare overall workload experience between the cued and voluntary tasks.

Finally, we conducted an exploratory analysis to examine if overall RT and switching cost differences between the cued and voluntary task (both in terms of tone-discrimination and naming RTs) differed depending on the type of naming approach participants followed in the voluntary task. Some people’s language use (and switching) might be entirely or largely driven by lexical access. In other words, they tend to use the word that comes to mind fastest, regardless of the language. Other people’s language choice might be driven by other variables too and might be less consistent. As a measure of how much participants used a lexically driven naming approach, we scored how consistently a given participant named each picture in either Mandarin or English in the voluntary task. For each item and participant, we scored the percentage of English language use relative to the number of accurate responses for that item. These percentages were recoded so that a score of .5 meant no consistency in language use (i.e., the item was named half of the time in English and half of the time in Mandarin by that participant) and 1 meant complete consistency (i.e., the item was always named in English or in Mandarin by that participant). For each participant, we then computed the mean consistency score across the 20 items. We included this variable (scaled and centred) in the analysis of both tone-discrimination and naming DVs to examine whether participants’ overall RT differences between tasks and switching costs were related to their naming-language consistency.

## Results

Accuracy was high in terms of both tone-discrimination (cued *M* = 93.9%, *SD* = 6.1; voluntary *M* = 96.5%, *SD* = 3.6) as well as picture naming (cued *M* = 94.7%, *SD* = 5.1; voluntary *M* = 99.0%, *SD* = 2.8). We therefore did not analyse accuracy further, but this confirmed that participants were completing both tasks as intended and paid attention. All participants included in the analyses met the pre-registered inclusion criteria of scoring at least 70% correct on the tone-discrimination task and the naming task and of having mean RTs in all conditions falling within 3 *SD* above/below the grand mean RT for each condition. All participants also produced switch and non-switch trials in both languages in the voluntary naming task. Switching frequency in the voluntary task was 33% (only including correct switch or non-switch trials; *SD* = 12.9, range = 8%–58%). English was used in 60% of the trials (*SD* = 13.3, range = 21%–92%). Switching frequency in Mandarin was 44% (*SD* = 15.6, range = 11%–77%), while it was 30% in English (*SD* = 14.5, range = 5%–58%).

### Tone discrimination (secondary task)

**Figure 2.**
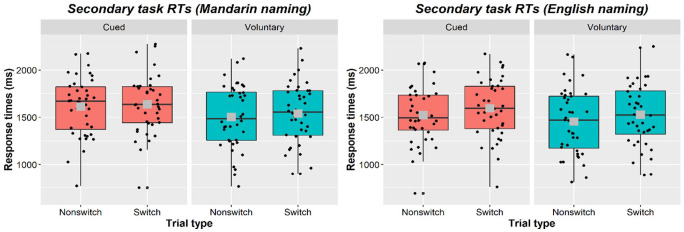
Box plots showing secondary-task responses by Language (left plot: Mandarin; right plot: English); Task (left panel within each plot: cued; right panel: voluntary); and Trial type (left box plot within each panel: non-switch; right plot: switch). Each black dot represents an individual participant’s mean. The horizontal black line represents the median while the centre of the grey square represents the overall mean.

The mean dual-tasking tone-discrimination RTs per condition are shown in Table 2 and Figure 2. There was a substantial dual-tasking cost of approximately 1 s relative to the single-task tone-discrimination mean RTs (*M single-task* RT cued session = 523, *SD* = 113; *M single-task* RT voluntary session = 545, *SD* = 118).

**Table 2.** Mean response times, RTs, (and SDs) in the tone-discrimination task by Task (cued or voluntary naming as the primary task), Language (naming in Mandarin or English), and Trial type (language switch or non-switch).

	Mandarin	English
Cued		
Non-switch	1,614 (312)	1,522 (301)
Switch	1,639 (305)	1,595 (311)
*Switching cost*	*25 (108)*	*74 (106)*
Voluntary		
Non-switch	1,503 (334)	1,455 (345)
Switch	1,540 (327)	1,527 (335)
*Switching cost*	*37 (110)*	*72 (163)*

The dual-task RTs showed a significant effect of task (β = 106.12, *SE* = 34.89, *t* = 3.042, *p* = .004), with overall responses being faster in the voluntary (*M* = 1,488, *SD* = 339) than cued (*M* = 1,590, *SD* = 298) task. RTs were also faster in the English condition (*M* = 1,505, *SD* = 307) than in the Mandarin condition (*M* = 1,583, *SD* = 282; β = −52.30, *SE* = 15.59, *t* = −3.354, *p* = .002). Finally, tone-discrimination single-task RTs were a significant predictor of RTs in the dual-task condition (β = 107.46, *SE* = 30.40, *t* = 3.535, *p* < .001).

There was a significant effect of trial type (β = 49.88, *SE* = 12.16, *t* = 4.100, *p* < .001), confirming the presence of a switching cost (see Table 2 and Figure 2). Importantly, trial type did not interact with task (β = −2.68, *SE* = 16.63, *t* = −.161, *p* = .873). The impact of switching languages on tone-discrimination RTs was comparable in the cued (*M switching cost* = 50, *SD* = 71) and voluntary task (*M switching cost* = 62, *SD* = 133). The switching cost was somewhat larger in English than in Mandarin (β = 41.07, *SE* = 20.23, *t* = 2.031, *p* = .050) but this was not modulated by task (β = 13.95, *SE* = 29.73, *t* = .469, *p* = .644). Language did not interact with task either (β = −29.86, *SE* = 20.80, *t* = −1.436, *p* = .160). This suggests neither Mandarin nor English switching costs differed between the two tasks.

### Naming task (primary task)

**Figure 3.**
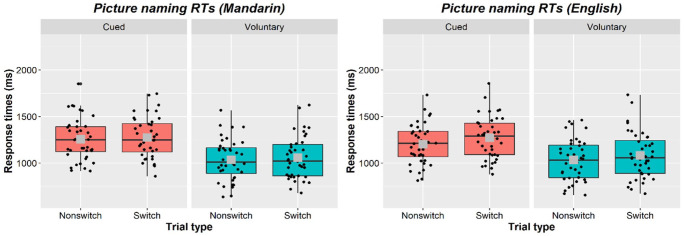
Box plots showing primary (naming) task responses by Language (left plot: Mandarin; right plot: English); Task (left panel within each plot: cued; right panel: voluntary); and Trial type (left box plot within each panel: non-switch; right plot: switch). Each black dot represents an individual participant’s mean. The horizontal black line represents the median while the centre of the grey square represents the overall mean.

Next, we analysed the naming onset times (see Table 3 and Figure 3). Similar to the tone RTs, there was a significant effect of task (β = 0.173, *SE* = 0.024, *t* = 7.247, *p* < .001). Overall naming was faster in the voluntary (*M* = 1046, *SD* = 227) than cued (*M* = 1250, *SD* = 214) task. There was a significant effect of trial type (β = .032, *SE* = 0.005, *t* = 6.648, *p* < .001), confirming the presence of a switching cost in the naming data too (see Table 3 and Figure 3). Importantly, trial type did not interact with task here either (β = .004, *SE* = 0.008, *t* = .548, *p* = .587). The switching cost for naming was comparable in the cued (*M* = 44, *SD* = 41) and voluntary task (*M* = 35, *SD* = 61).

**Table 3.** Mean naming response times, RTs, (and SDs) in the picture-naming task by Task (cued or voluntary), Language (Mandarin or English), and Trial type (language switch or non-switch).

	Mandarin	English
Cued		
Non-switch	1,257 (226)	1,203 (214)
Switch	1,274 (219)	1,272 (226)
*Switching cost*	*18 (85)*	*69 (75)*
Voluntary		
Non-switch	1,035 (220)	1,033 (222)
Switch	1,058 (230)	1,083 (247)
*Switching cost*	*23 (78)*	*49 (77)*

Note that the picture was presented 300 ms before the tone presentation; the naming times above are computed relative to the onset of picture presentation.

The switching cost for naming was larger in English than in Mandarin (β = .030, *SE* = 0.012, *t* = 2.418, *p* = .021), but this was again not modulated by task (β = .018, *SE* = 0.018, *t* = .995, *p* = .326). There was no main effect of language^1^ (β = −.007, *SE* = 0.010, *t* = −.706, *p* = .484), but in terms of naming RTs, language did interact with task (β = −.028, *SE* = 0.013, *t* = −2.243, *p* = .031). In the cued task, naming was significantly faster in English than in Mandarin (see Table 3; β = −.021, *SE* = 0.009, *t* = −2.276, *p* = .028), while the voluntary task showed no significant language effect (β = .008, *SE* = 0.009, *t* = .867, *p* = .392).^2^

### NASA workload experience

Next, we compared how participants evaluated experienced workload in the cued and voluntary tasks. Overall workload was experienced to be higher in the cued task (*M* = 55, *SD* = 18) than in the voluntary task (*M* = 45, *SD* = 17; *t*(39) = 3.356, *p* = .002, *d* = .531).

### Naming consistency in the voluntary task (exploratory analysis)

As an exploratory analysis, we also examined whether both secondary tone RTs and primary task naming times differed depending on the approach participants used in the voluntary task. This analysis included a participant’s naming consistency as an additional predictor, with higher scores reflecting that a participant was more consistent in the language they named a given item in. The secondary-task tone RTs showed no main effect of naming consistency (β = −38.94, *SE* = 45.42, *t* = −.857, *p* = .397), but naming consistency did interact with task (β = 77.17, *SE* = 33.18, *t* = 2.326, *p* = .025). Cued task RTs were not related to voluntary naming consistency (β = 10.49, *SE* = 49.14, *t* = .214, *p* = .832). The relationship was not significant in the voluntary task either (β = −76.92, *SE* = 46.04, *t* = −1.671, *p* = .103), but the direction showed that participants with higher naming consistency were generally faster overall in the voluntary task than those with lower naming consistency. The significant interaction with task thus shows that the overall RT difference observed between the cued and voluntary task (with faster responses in the voluntary task) was largest for people adopting a consistent voluntary naming approach, which was confirmed by a correlation between naming consistency and a participant’s RT difference between cued and voluntary tone RTs (*r* = .382, *p* = .015). The same pattern was observed in the naming RT data, but the interaction between naming consistency and task did not reach significance (β = .039, *SE* = 0.024, *t* = 1.669, *p* = .103).

Finally, we assessed whether there was a relationship between naming consistency and switching costs across the two tasks. No such relationship was found; naming consistency was not related to switching costs in the tone data (trial type × naming consistency: β = −5.34, *SE* = 12.49, *t* = −.427, *p* = .672; trial type × naming consistency × task: β = 12.67, *SE* = 17.10, *t* = .741, *p* = .464). Similarly, naming consistency was not related to switching costs in the naming RT data (trial type × naming consistency: β = .001, *SE* = 0.005, *t* = .265, *p* = .793; trial type × naming consistency × task: β = .004, *SE* = 0.008, *t* = .532, *p* = .598). This suggests that while the overall cued versus voluntary RT difference was related to voluntary naming consistency, switching costs (which were similar for the cued and voluntary task) were not related to naming consistency.

## Discussion

This study assessed potential differences in attentional resource demands between cued and voluntary language production, both in terms of more proactive and sustained mechanisms (overall differences between the tasks affecting non-switch trials too) as well as reactive mechanisms related to language switching (switching costs across tasks). Participants completed a tone-discrimination task while naming pictures in response to cues or freely in their language of choice. Tone-discrimination RTs differed between the two naming tasks, with faster responses while completing the voluntary task than the cued task simultaneously. The NASA task load index also showed that participants experienced the cued dual-task paradigm to be more demanding in terms of overall workload than the voluntary dual-task paradigm, despite no differences in the tone discrimination task requirements. Longer RTs were also found in trials involving a language switch from the preceding trial (i.e., a “switching cost”). However, this switching cost did not differ between the cued and voluntary task.

### Overall differences between the cued and voluntary task

Secondary task (tone) RTs were faster when participants named pictures in their language of choice instead of in response to cues. This suggests the cued task was more effortful and placed higher demands on attentional resources, thus leaving fewer resources to complete the secondary task. These findings were in line with the naming data, which also showed faster voluntary than cued naming RTs. Participants also reported a higher level of overall workload during the cued than voluntary task. Together, these findings strongly suggest that the cued task is associated with higher levels of overall control, in line with the Adaptive Control Hypothesis (Green & Abutalebi, 2013), likely mostly related to proactive and sustained control as these effects were observed across switch and non-switch trials. These findings also align with previous literature finding faster voluntary than cued naming times (e.g., de Bruin et al., 2018; Gollan et al., 2014; Jevtović et al., 2020) and cued mixing *costs* but voluntary mixing *benefits* (e.g., de Bruin et al., 2018; Jevtović et al., 2020).

These discrepant control demands are likely to stem from various differences between the two types of language-use environments (Green & Abutalebi, 2013). As a first step, cued language use requires participants to detect cues to know which language to use. We ensured the two tasks were comparable in terms of visual cue input by including the same (combination of) visual cues in each task. However, the cues were naturally more relevant for the task in the cued context and as such required deeper processing. As a next step, a cued context requires overall goal maintenance, to ensure the language associated with the cue is used at the appropriate time, as well as overall conflict monitoring and interference suppression to avoid interference from the non-target language. Bilinguals might proactively balance the two languages through inhibition of the dominant language (Green, 1998) and/or (over-)activation of the less-dominant language (Philipp et al., 2007) to allow for flexible use during a cued naming task. The slower tone-discrimination task responses in the cued than voluntary condition seem likely to reflect this more sustained, proactive language control and increased recruitment of attentional resources in anticipation of cued naming, forming the initial stages of language production. Participants were asked to prioritise the picture naming task, but appear to have, in some instances, prioritised the tone-discrimination task instead. For example, in some trials, participants responded to the tone before they started to actually name the picture. On these trials too, however, they had already seen the picture and cue for 300 ms as these were always presented 300 ms before the tone. Even on the trials where participants responded to the tone before naming the picture, responses were faster in the voluntary than cued condition (*M voluntary* = 893 ms versus *M cued* = 987 ms, including only the 10 participants who occasionally did this on both tasks^3^). This again suggests the initial stages of cue detection, goal maintenance, and to some extent language/word selection prior to actual production, used more attentional resources in the cued than voluntary task.

The cued and voluntary tasks differed in the overall switching frequency as voluntary switching frequency was determined based on the participants’ responses and not controlled by the design. Exploratory analyses (see Footnote 2) showed that participants who switched more often during voluntary naming responded more slowly in the tone-discrimination task. Lower voluntary switching rates could be part of the reason why overall tone-discrimination RTs were faster in the voluntary than cued task. However, similar cued-voluntary RT differences were found in terms of naming latencies too, which did not show a significant relationship with switching frequency. Furthermore, after including switching frequency in the analyses, the main effect of task remained present in both the tone-discrimination RTs and the naming latencies. This suggests the observed task effects cannot be (entirely) ascribed to switching frequency.

The faster voluntary RTs might thus be the consequence of the voluntary task posing lower attentional control demands but could also be the result of the voluntary task benefitting from more opportunistic language use that allows bilinguals to use the words that come to mind fastest, regardless of the language. Indeed, the difference between overall tone-discrimination RTs in the cued versus voluntary task was also associated with *how* bilinguals used their languages in the voluntary task. Participants who used their languages more consistently for each item (e.g., always or often naming a certain picture in English OR in Mandarin) showed a larger difference between cued and voluntary tone-discrimination RTs. While the relationship within the voluntary task itself did not reach significance, this interaction was driven by voluntary overall RTs being faster when the languages were used more consistently. A similar pattern was observed in the naming data but did not reach significance. An important factor in voluntary language choice is how quickly participants can retrieve a word in each language (e.g., de Bruin et al., 2018). Those who consistently follow the same language for each item might benefit most from always (or often) producing the word that comes to mind fastest, and as such might show a larger difference between the voluntary and cued task. Indeed, both language choice and naming consistency were found to be strongly related to how fast participants could name each item in each language. Further exploratory checks were conducted based on the practice round in which participants named each item once in each language for the first time. Participants who showed a larger RT difference between languages on a given item in this practice phase were also more likely to consistently name the item in the same language in the voluntary naming task. In the current study, that relationship between naming consistency and baseline RT differences between languages was found to be related to item-specific RTs rather than more global RT differences between languages. However, it is likely that naming consistency is also related to overall language dominance, with participants more frequently using their overall stronger (i.e., faster) language.

In contrast, those who do not fully follow such a consistent naming approach might benefit less from faster retrieval and might also apply more proactive control during voluntary naming. In these cases, bilinguals might choose their languages in response to other variables that are not directly related to lexical access and require more cognitive resources to implement language choice in a more controlled, top-down manner. For instance, bilinguals might prefer to stay in the same default language throughout or might have a strong global preference for a specific language that can overrule lexical-access advantages in the other language. They might also be more strongly influenced by the environment or languages used on previous items. These different factors might result in more top-down-driven language choice requiring more control.

### Cued versus voluntary switching

Language switching had an impact on secondary task tone-discrimination RTs, which were longer when participants had to switch languages. This showed that dual-task paradigms are sensitive to resource demands used during language switching, similar to other language production tasks (e.g., Piai & Roelofs, 2013). However, this switching cost did not differ between cued and voluntary naming in terms of either secondary-task tone-discrimination RTs or primary-task naming RTs. This suggests that both types of switching recruited additional attentional resources relative to continuing to use the same language. This is in line with several other studies who have found similar cued and voluntary switching costs (e.g., de Bruin et al., 2018; Gollan et al., 2014). Other studies have also found voluntary switching costs, although of a smaller size than cued costs (e.g., de Bruin & Xu, 2023; Gollan et al., 2014). Together with these studies, the current study suggests that voluntary switching might also require cognitive effort, even though the switch is initiated by the bilinguals themselves. This finding contrasts with other studies that show no voluntary language switching costs (e.g., Zhu et al., 2022), although this appears to be mostly the case in situations in which language choice is completely or mostly driven by lexical access.

In daily-life language switching, however, there might be a wealth of reasons why bilinguals switch voluntarily, and reactive language control might not be suspended entirely. It is possible that even cooperative language control (Green & Wei, 2014) requires a certain degree of control to make sure a given word is only produced in one language at a time. With both languages being active (and continuing to compete for selection), there might be some ongoing interference from the other language, even if the word can be retrieved faster in the used language. Furthermore, when switching languages, the relative competition from the “other” language (used in the previous trial) might be higher than in non-switch trials. While this interference from the previous trial might be smaller during voluntary than cued switching (de Bruin & Xu, 2023), it is possible that a certain amount of language competition and interference remains even during voluntary switching. This might require inhibition, and potentially also additional time to overcome previously applied inhibition. Indeed, in the current study, bilinguals showed larger costs when switching to English than to Mandarin, in both the cued and voluntary task. While Mandarin was the participants’ first language in terms of Age of Acquisition (although some participants also acquired English from birth), participants were living in the United Kingdom or United States and English was the most-used language in their daily lives. Asymmetrical switching costs (with larger costs when switching back to the more dominant language) are often interpreted as a reflection of the amount of inhibition used during language switching (Green, 1998). When using Mandarin, participants might have suppressed English (the more dominant language) more strongly. Consequently, when switching back to English, they might have needed additional time to release this previously applied inhibition, thus leading to larger switching costs. The presence of these asymmetrical costs in both cued and voluntary switching tasks could suggest that reactive language control (possibly in the form of inhibition) over the language that is not currently used continued to play a role in both types of switching contexts.

While the type of language use (naming language consistency) influenced overall RTs, it was not related to switching costs. Previous studies that manipulated language consistency through instructions and/or stimuli used (Kleinman & Gollan, 2016; Zhu et al., 2022) found no significant voluntary switching costs. However, studies using more neutral stimuli and no specific instructions have found more mixed findings when assessing individual differences in naming consistency. For example, Gollan et al. (2014) found an association between switching costs and naming consistency, while de Bruin et al. (2018) found a relationship with the mixing benefit but not the switching cost. The current study suggests that using a more consistent naming language, likely reflecting a more bottom-up lexically driven approach, might have a positive impact on *both* non-switch and switch trials. In other words, consistently using the same word might allow a bilingual to benefit more from faster naming *constantly*, not just when a language switch is made. This is in line with recent findings by de Bruin and Martin (2022), who showed that using a language that aligns with an individual’s personal language choices was related to faster overall responses but did not influence switching costs in particular.

### Proactive versus reactive language control

Following the Adaptive Control Hypothesis (Green & Abutalebi, 2013), dual- and free-switching environments are expected to differ in terms of proactive and reactive control. Cued and voluntary picture-naming tasks, although they measure word production in isolation and not in sentence contexts or in dialogue with a communication partner, are most closely related to dual-language and free-switching environments, respectively. Our findings support the Adaptive Control Hypothesis in demonstrating proactive control differences. The dual-tasking data strongly suggest that using two languages in dual-language environments requires more attentional resources than freely using two languages, which benefits from a combination of fewer attentional demands (e.g., lower demands regarding cue processing and goal maintenance) and more opportunistic language use. These voluntary benefits during dual-tasking furthermore suggest that bilinguals do not deliberately reflect upon which language they use for each item (or at least that they do not spend any attentional resources on this relative to cued naming), although this might occur when bilinguals are asked to explicitly indicate what their language choice is (Reverberi et al., 2018).

In contrast, our study does suggest attentional resources are recruited during voluntary language switching. This suggests that some form of reactive control demands might remain present even during more cooperative control (Green & Wei, 2014). While the current study showed that dual-tasking performance was similar during cued and voluntary switching, this similarity in behavioural performance does not have to reflect (similar) underlying control mechanisms related to reactive control. It might reflect active control mechanisms used to manage interference at the actual moment of switching from the “other language” but might also reflect competition between languages, which could be higher when the other language has just been used. However, our data suggest that the switching cost as such cannot be explained by the extent to which participants’ language use is consistent and potentially driven by lexical access. It therefore leaves open the possibility that all bilinguals use some degree of control at the actual moment of language switching.

## Conclusion

In daily life, bilinguals might switch languages for a wealth of reasons. However, most psycholinguistic research has focussed on assessing language switching in response to cues. This overlooks a very common type of language switching in which language choice is more volitional (e.g., when conversing with other bilinguals who speak the same languages). Here we show that different types of language use (specifically, cued versus free naming) pose different attentional control demands. Rather than focusing solely on differences in naming times, we assessed potential differences in terms of attentional resources used during a dual-tasking paradigm. The moment of language switching was associated with a switching cost, regardless of whether that switch was made voluntarily or in response to a cue. This suggests that reactive control is not suspended entirely during voluntary language switching. However, overall, voluntary language use required fewer attentional resources than cued language use, and this was reflected in both behavioural and subjective markers of cognitive effort. This suggests that while there might still be a cost associated with the actual moment of switching, freely choosing how to use both languages reduces effort compared to adjusting language use to environmental cues.

## Supplemental Material

sj-docx-1-qjp-10.1177_17470218231173638 – Supplemental material for Dual-tasking while using two languages: Examining the cognitive resource demands of cued and voluntary language production in bilinguals Supplemental material, sj-docx-1-qjp-10.1177_17470218231173638 for Dual-tasking while using two languages: Examining the cognitive resource demands of cued and voluntary language production in bilinguals by Angela de Bruin and Ronan McGarrigle in Quarterly Journal of Experimental Psychology

## Notes

1 Mandarin is a tonal language and we therefore checked whether Mandarin was influenced more by the tone-discrimination task than English. We compared the naming times in the dual-task parts to the single-task naming practice phase without tones. There was no interaction between language and dual-tasking, suggesting Mandarin was not affected more by the secondary tone-discrimination task (although it should be noted that the presence of the secondary task was confounded with single- or dual-language naming contexts in this check).

2 Switching frequency was lower in the voluntary than the cued task. We therefore checked whether voluntary switching frequency modulated the results assessing differences between cued and voluntary tasks as reported. Switching frequency was related to tone-discrimination RTs during the voluntary tone-discrimination task (participants who switched less often responded faster, *r* = .399, *p* = .011) but was not significantly associated with tone-discrimination switching costs (*r* = −.266, *p* = .097), nor with overall naming RTs (*r* = .087, *p* = .594) or naming switching costs (*r* = −.117, *p* = .470). In line with the correlational analyses, including switching frequency in the tone-discrimination model showed an interaction between frequency and task (*p* = .023). This was driven by participants who switched more often in the voluntary task also responding more slowly to tones in the voluntary condition (*p* = .051). However, the naming RT models showed no interaction between task and switching frequency (*p* = .330). Importantly, including voluntary switching frequency in all models showed the same key results as the models reported in sections “Tone discrimination (secondary task)” and “Naming task (primary task).”

3 Five of these participants frequently reversed the order of tasks in their responses (>20% of trials). Removing these participants entirely did not change the results.
