# Supplementary material for: Dual-tasking while using two languages: Examining the cognitive resource demands of cued and voluntary language production in bilinguals
Source: Q J Exp Psychol (Hove). 2023 May 18;77(3):461–77. doi: 10.1177/17470218231173638 (PMC10880414; doi:10.1177/17470218231173638)
Supplement: sj-docx-1-qjp-10.1177_17470218231173638 – Supplemental material for Dual-tasking while using two languages: Examining the cognitive resource demands of cued and voluntary language production in bilinguals [file sj-docx-1-qjp-10.1177_17470218231173638.docx]

Supplementary Material for:

**Dual-tasking while using two languages: Examining the cognitive resource demands of cued and voluntary language production in bilinguals**

Angela de Bruin^1^ & Ronan McGarrigle^2^

^1^Department of Psychology, University of York, York, UK

^2^Department of Psychology, University of Bradford, Bradford, UK

Corresponding author:

Angela de Bruin

Department of Psychology

University of York

York YO10 5DD

UK

angela.debruin@york.ac.uk

*Mandarin and English words used in the Experiment.*

| **Mandarin** | **English** |
| --- | --- |
| 猫 | cat |
| 鸭子 | duck |
| 苹果 | apple |
| 房子 | house |
| 小船 | boat |
| 钢琴 | piano |
| 眼睛 | eye |
| 帽子 | hat |
| 奶牛 | cow |
| 狐狸 | fox |
| 狗 | dog |
| 狮子 | lion |
